# Responding to COVID19: What is happening for our vulnerable children and young people?

**DOI:** 10.1017/cha.2020.18

**Published:** 2020-04-24

**Authors:** Jennifer Lehmann

**Affiliations:** Social Work, La Trobe Rural Health School, La Trobe University, Bendigo, Victoria, Australia

**Keywords:** COVID19, children and young people, pandemic responses

As I write this in mid-April 2020, our world has and is continuing to change with concerns about fire and climate change giving way to additional concerns that have flowed from the declaration of the COVID19 pandemic and the measures instituted in many countries in the world. We have not seen an infection that is so far-reaching for over a century, and the impacts have been significant. Families have been affected socially and economically, millions have lost their income, some have lost their homes and with some 165,000 people dead at the time of writing, so many people are affected by the loss of loved ones and the grief of not being with them at the time of their deaths. Adults have rallied and found ways of expressing their agency in these difficult times with social distancing supported, for many, through our contemporary communication technology. So many creative approaches to staying in touch have emerged, so many people have found amusing ways of expressing their experiences of lockdowns and home isolation. But there are also thousands of vulnerable children and young people who had limited agency and no voice before the pandemic. What is happening for them as they confront the stresses and increased disadvantage likely as a result of COVID19? What about our children in foster care and in residential care; the children of asylum seekers and refugees, children exposed to what has been described as a ‘spike’ in family violence (Jolie, 2020; Neuman, 2020; O’Donnell et al., 2020) and many others?

It has been heartening to find that many of our international and national welfare associations have swiftly responded with information on COVID19, together with some practical responses. End Violence Against Children (2020) has published a webpage titled ‘Protecting children during the Covid-19 outbreak’ which provides a range of information and important international links. One of these leads to Save the Children (2020) which has swiftly developed a learning package titled: The COVID-19 Learning Pathway which contains the following: ‘Online technical capacity strengthening programmes to support humanitarians’ responses during this crisis, covering a number of critical topics, including Public Health, Child Protection and Gender/Equality. Online soft skills and remote working capacity strengthening programmes to support humanitarians’ responses during this crisis’ and ‘A library of key downloadable resources relating to working in the context of COVID-19, including remote working guides and resilience support’ (n.p.). And The Child Protection Hub for South-East Europe has also developed a support site with numerous resources including for families, communities, and for children.

Health and Education websites have also been quick to respond with information. For instance, The Department for Education and Public Health England (2020) are giving priority to vulnerable children, recognizing that many are protected by attending school and making provision for schools to have these children attend stating: ‘Leaders of educational settings and designated safeguarding leads know who their most vulnerable children are and will have the flexibility to offer a place to those on the edges of receiving children’s social care support’ and ‘There is an expectation that vulnerable children who have a social worker will attend an education setting, so long as they do not have underlying health conditions that put them at severe risk. In circumstances where a parent does not want to bring their child to an education setting, and their child is considered vulnerable, the social worker and education setting should explore the reasons for this, directly with the parent’ (n.p.).

In the USA, schools are closed to the end of the American school year (June 2020). Paul Reville, former secretary of education for Massachusetts, interviewed by the Harvard Gazette reporter, Liz Mineo, commented:

Children come from very different backgrounds and have very different resources, opportunities, and support outside of school. Now that their entire learning lives, as well as their actual physical lives, are outside of school, those differences and disparities come into vivid view. Some students will be fine during this crisis because they’ll have high-quality learning opportunities, whether it’s formal schooling or informal homeschooling of some kind coupled with various enrichment opportunities. Conversely, other students won’t have access to anything of quality, and as a result will be at an enormous disadvantage. Generally speaking, the most economically challenged in our society will be the most vulnerable in this crisis, and the most advantaged are most likely to survive it without losing too much ground (Mineo, 2020).

The more introverted children, so long as they have not experienced disadvantage, are probably able to learn well under the school closure and online learning regime. These are the children often forgotten in our extrovert world. They are the quiet ones who find noisy groups disturbing and distracting, and they certainly do not learn well in crowded situations, nor in the contemporary classrooms that house more than one class simultaneously with two teaching staff, the majority of whom tend to be extroverted. Laney (2005) describes the school setting as often challenging for introverted children and young people who may prefer the library to the playground to recoup energy during break times. They find the close proximity, noise, interruptions, and general rush of the classroom energy sapping and learn better when there is a quiet atmosphere, privacy, and lack of pressure to respond (Laney, 2005).

However, temperament type and learning style are not the only drivers of learning and success in education, and for many disadvantaged children, there have been obstacles to even getting started in their educational careers. Children and young people who are in foster care, residential care, or in correctional institutions often struggle in the educational context. And what about the children of asylum seekers and refugees whose lives have been constantly disrupted? These groups of children and young people are the most vulnerable and disadvantaged members of society. Their educational achievements are often not strong because of them facing disruption in their lives, so being schooled online at home may not suit them well.

CoramBAAF (2020) and The Fostering Network, UK (2020) developed guides for foster parents, together with support links, whose children were or are at home during the lockdown period. Other such networks have also been quick to provide guidance including the Centre for Excellence in Child and Family Welfare in Victoria (Australia), and in India, the Ministry of Women and Child Development has issued information for carers of children. The response, if sometimes somewhat general in description in some cases, has certainly been swift, but it depends on people having Internet and telephone access as well as other avenues for gaining support at a more personal level. The latter is not necessarily straightforward when social distancing is required, though there has been a huge upsurge in the use of video linking via Zoom, Microsoft Teams, WhatsApp, and the like.

Telehealth services have also been quick to emerge and may outlast the COVID19 pandemic as a stronger means for communicating with parents of vulnerable children as well as people who are often required to travel significant distances to obtain specialist assistance for what is often just a few minutes of the busy specialist’s time. Telehealth is not new and have been steadily increasing in some health systems, but Topol (2020), writing for *The Economist*, believes it will be a legacy of the COVID19 pandemic and is particularly important for people living remotely. He states, while telehealth ‘also needs to overcome regulatory and commercial hurdles and requires a digital infrastructure that ensures secure connections between patients and physicians’ that ‘Telemedicine will play the role of the first consultation’ in the future (Topol, 2020 n.p.). Both Australia and the USA have instituted telehealth and phone consultation regimes, and this eases the pressure on all parents coping with children at home, not just those caring for foster children or children with disability, or living remotely. In fact, Imenokhoeva (2020, n.p.) writes that ‘Today we are looking for the best practices and implemented solutions from around the globe. The USA, Israel, UK, Nordics and France are among the leading countries with mature telehealth providers, and their cumulative experience is helping them to respond to the current situation.’ This agility in the use of technology will be of significant benefit in general health and medicine as well as during the pandemic and perhaps sets part of the ‘new normal’ many of us expect to emerge.

For young people in residential care or correctional facilities, the pandemic may not be so easy to manage, though in every state in Australia guidelines have been issued by the relevant government department. Once the infection is introduced into a residence or other facility, it is difficult to see how others would not quickly become infected. Young people are reliant on care staff to ensure that they are referred, tested, and treated swiftly, but issues with lack of trust by young people and oppositional behaviors may well affect the response they receive. The Better Care Network, in association with The Alliance for Child Protection in Humanitarian Action and UNICEF (2020), has published an online document with links relating to specific issues, and this, if followed, would go some way in ensuring that staff and carers are informed. However, it is also hoped that the pandemic will, indeed, be less damaging to children and young people as has been suggested, though for vulnerable young people with compromised health this is a risky supposition.

This brief commentary gives an overview of some of the efforts being made in the child and youth sector to protect vulnerable young people and support their carers during the period of the pandemic. However, it must be acknowledged that in countries where the death toll has been significant, there are going to be many children and young people who are grappling with loss and grief on top of their already challenging circumstances. There are no easy answers, but it is hoped that staff and carers working with them are able to rise to the emergency and provide a high standard of interaction and care that goes some way to helping them overcome their disadvantage.
